# Prognostic impact of *ATM* mutations in patients with metastatic colorectal cancer

**DOI:** 10.1038/s41598-019-39525-3

**Published:** 2019-02-27

**Authors:** Giovanni Randon, Giovanni Fucà, Daniele Rossini, Alessandra Raimondi, Filippo Pagani, Federica Perrone, Elena Tamborini, Adele Busico, Giorgia Peverelli, Federica Morano, Monica Niger, Maria Antista, Salvatore Corallo, Serena Saggio, Beatrice Borelli, Gemma Zucchelli, Massimo Milione, Giancarlo Pruneri, Maria Di Bartolomeo, Alfredo Falcone, Filippo de Braud, Chiara Cremolini, Filippo Pietrantonio

**Affiliations:** 1 Medical Oncology Department, Fondazione IRCCS Istituto Nazionale dei Tumori di Milano, Via G. Venezian, 1 - 20133 Milan, Italy; 2 Unit of Medical Oncology 2, Azienda Ospedaliero-Universitaria Pisana, Via Roma, 67 - 56126 Pisa, Italy; 3 Department of Pathology and Laboratory Medicine, Fondazione IRCCS Istituto Nazionale dei Tumori di Milano, Via G. Venezian, 1 - 20133 Milan, Italy; 4 Department of Oncology and Hemato-Oncology, University of Milan, Via Festa del Perdono, 7 - 20122 Milan, Italy; 5 Department of Translational Research and New Technologies in Medicine and Surgery, University of Pisa, Via Risorgimento, 36 - 56126 Pisa, Italy

## Abstract

Tumors bearing homologous recombination deficiency are extremely sensitive to DNA double strand breaks induced by several chemotherapeutic agents. *ATM* gene, encoding a protein involved in DNA damage response, is frequently mutated in colorectal cancer (CRC), but its potential role as predictive and prognostic biomarker has not been fully investigated. We carried out a multicenter effort aimed at defining the prognostic impact of *ATM* mutational status in metastatic CRC (mCRC) patients. Mutational profiles were obtained by means of next-generation sequencing. Overall, 35 out of 227 samples (15%) carried an *ATM* mutation. At a median follow-up of 56.6 months, patients with *ATM* mutated tumors showed a significantly longer median overall survival (OS) versus *ATM* wild-type ones (64.9 vs 34.8 months; HR, 0.50; 95% CI, 0.29–0.85; *P* = 0.01). In the multivariable model, *ATM* mutations confirmed the association with longer OS (HR, 0.57; 95% CI, 0.33–0.98; *P* = 0.04). The prognostic impact of *ATM* mutations was independent from *TP53* mutational status and primary tumor location. High heterogeneity score for *ATM* mutations, possibly reflecting the loss of wild-type allele, was associated with excellent prognosis. In conclusion, we showed that *ATM* mutations are independently associated with longer OS in patients with mCRC.

## Introduction

Significant advances in the implementation of biomarkers in the clinical practice have been achieved in metastatic colorectal cancer (mCRC), even if only few of them (such as *RAS* and *BRAF* mutational status or microsatellite instability [MSI]) are endowed with clinical relevance. Furthermore, despite the advances achieved in understanding the molecular bases of resistance to EGFR targeting agents^1–3^, there is still a lack of biomarkers able to predict sensitivity/resistance to chemotherapy, which remains the cornerstone of treatment for most patients.

Cancer cells may gain the potential for uncontrolled growth by escaping functional cell-cycle checkpoints. By doing so, they simultaneously become vulnerable to both endogenous (*e*.*g*. oncogenic-driven replication stress) and exogenous (*e*.*g*. DNA-damaging agents) genotoxic insults^4^. Tumors with homologous recombination deficiency are extremely sensitive to cross-linking agents such as platinum salts, or topoisomerase inhibitors. This mechanism has substantial implications in the clinical practice, specifically concerning the management of those tumors bearing deleterious *BRCA1-2* mutations (*e*.*g*. *BRCA*-mutated breast and ovarian cancer)^5,6^.

*Ataxia-Telangiectasia Mutated* (*ATM*) is a gene member of the highly conserved PI3K-related kinases, on which cells rely for orchestrating the DNA damage response (DDR) for both DNA repair and cell-cycle checkpoint activation. Specifically, ATM is recruited upon DNA double strand breaks (DSBs) and is involved in DNA repair via both BRCA1-driven homologous recombination and non-homologous end-joining pathways, as well as in the G1/S cellular checkpoint activation through its major targets p53 and CHK2^7^.

Germline and somatic mutations involving homologous recombination related genes, including *ATM*, are predicted to confer an enhanced platinum sensitivity^8^. Specifically, ATM deficient tumors display a higher sensitivity to DNA DSB-inducing treatments^9^ and loss of function mutations affecting the *ATM* gene could confer a vulnerability to DNA-damaging agents, especially in combination with p53 deficiency^10–13^. Because of the consistent prevalence of *ATM* mutations in CRC (7% in non-hypermutated cases)^14^ and their potential crucial role as biomarker of chemosensitivity to platinum salts and topoisomerase inhibitors, *ATM* mutations would therefore characterize mCRC patients with a more favourable outcome, at least when eligible for combination chemotherapy. Moving from this background, we performed a translational study aimed at assessing the prognostic relevance of *ATM* mutational status in mCRC patients.

## Materials and Methods

### Patients population

We retrieved pre-treatment tumor tissue blocks of initially unresectable mCRC patients treated at two Italian Institutions (Fondazione IRCCS Istituto Nazionale dei Tumori di Milano and Azienda Ospedaliero-Universitaria Pisana). Clinical, pathological and molecular characteristics at the time of diagnosis of metastatic disease were collected, including age, gender, Eastern Cooperative Oncology Group (ECOG) Performance Status (PS), primary tumor location (right- vs left-sided), primary tumor resection (yes vs no), time-to-metastases (synchronous vs metachronous), number of metastatic sites (1 vs >1), *RAS* and *BRAF* mutational status, and MSI status. All included patients received at least one treatment line with doublet or triplet regimens with or without monoclonal antibodies according to standard clinical practice. The study was approved by the Fondazione IRCCS Istituto Nazionale dei Tumori di Milano Institutional Review Board (study ID: INT 117/15) and conducted according to the ethical principles for medical research involving human subjects adopted in the Declaration of Helsinki. All patients signed a written informed consent.

### Next-generation sequencing analysis

We centrally collected formalin-fixed paraffin-embedded archival tumor tissue blocks. Next-generation sequencing (NGS) data were obtained through the Ion AmpliSeq Cancer Hotspot Panel v2 (Life Technologies) with the Ion-Torrent™ Personal Genome Machine platform (Life Technologies), as previously described^15,16^ and detailed in Supplementary Methods (see Supplementary Information). *ATM* and *TP53* mutational status was obtained, and *RAS* and *BRAF* mutational status was centrally confirmed. Heterogeneity score (HS) of *ATM* mutations was calculated as previously described by Normanno *et al*.^17^. Briefly, the mutant allelic frequency was normalized for the neoplastic cell content, and the HS was calculated by multiplying by 2 the frequency of mutant alleles in neoplastic cells as somatic mutations usually involve only one allele.

### Statistical analysis

Chi-square test or fisher exact test were used, as appropriate, to evaluate the association between *ATM* mutational status and the other relevant clinical and pathological patients’ characteristics. Overall survival (OS) was calculated as the time from diagnosis of metastatic disease to the death from any cause. Since chemotherapy sensitivity putatively caused by *ATM* mutations may be boosted by the concomitant presence of *TP53* mutations^10^ or primary tumor sidedness due to enrichment of mesenchymal and stem-like subtypes in right-sided tumors^18^ we also evaluated the prognostic impact of combined *ATM* and *TP53* mutational status assessment as well as the prognostic impact of combined *ATM* mutational status and primary tumor location. The Kaplan-Meier method and the Cox proportional-hazards model were used for survival analyses. Hazard ratios (HRs) together with 95% confidence intervals (CI) were provided. Statistical significance threshold was set to a two-tailed 0.05 value. R software (version 3.5.0) and RStudio software (version 1.1.453) were used for Statistical analyses.

## Results

### Clinical, pathological and molecular features of *ATM* mutated mCRC

As detailed in the Consort diagram (Supplementary Fig. S1 in Supplementary Information), the final study population included a total of 227 patients, of whom 35 (15%) had *ATM* mutated tumors and 192 (85%) *ATM* wild-type tumors. *TP53* mutations were found in a total of 148 (65%) of samples, of whom 24 (69%) in the *ATM* mutated subgroup and 124 (65%) in *ATM* wild-type one (*P* = 0.65). Table 1 shows patients’ demographics and disease characteristics, overall and according to *ATM* mutational status. Of note, *ATM* mutations were not significantly associated with specific clinical and molecular features. The exposure to specific agents approved for mCRC and the number of treatment lines received are summarized in Supplementary Table S1 (see Supplementary Information). Table 2 illustrates the specific mutations found in *ATM* gene and concomitant “trunk” mutations affecting *TP53*, *KRAS*, *NRAS*, *BRAF* and *APC*, with relative HS. The median HS for *ATM* mutations was 116 (IQR, 51–197).

**Table 1:** Patients’ and disease characteristics, overall and according to *ATM* mutational status.

Characteristics		Total (N = 227) N (%)	*ATM* mut (N = 35) N (%)	*ATM* wt (N = 192) N (%)	P*
Age (years)	<65 ≥65	147 (65) 80 (35)	25 (71) 10 (29)	122 (64) 70 (36)	0.40
Gender	Male Female	93 (41) 134 (59)	17 (49) 18 (51)	76 (40) 116 (60)	0.32
ECOG PS	0 1–2 NA	197 (92) 18 (8) 12	34 (97) 1(3) 0	163 (91) 17 (9) 12	0.20
Primary tumor location	Left-sided Right-sided	159 (70) 68 (30)	27 (77) 8 (23)	132 (69) 60 (31)	0.32
Primary tumor resection	Yes No	192 (85) 35 (15)	31 (89) 4 (11)	161 (84) 31 (16)	0.48
Synchronous mets	No Yes	67 (30) 160 (70)	13 (37) 22 (63)	54 (28) 138 (72)	0.28
Metastatic sites (N)	1> 1	135 (59) 92 (41)	24 (69) 11 (31)	111 (58) 81 (42)	0.23
All-*RAS* status	Wild-type Mutated	127 (56) 100 (44)	20 (57) 15 (43)	107 (56) 85 (44)	0.88
*BRAF* status	Wild-type Mutated	214 (94) 13 (6)	33 (94) 2 (6)	181 (94) 11 (6)	0.99
MSI status	MSS MSI NA	188 (94) 13 (6) 26	26 (87) 4 (13) 5	162 (95) 9 (5) 21	0.11

*Chi-square test or Fisher exact test, as appropriate.

*Abbreviations*. ECOG PS: Eastern Cooperative Oncology Group Performance Status. MSI: microsatellite instability. MSS: microsatellite stability. Mut: mutated. Wt: wild-type.

**Table 2:** Specific mutations found in *ATM* gene with concomitant “trunk” mutations (affecting *TP53*, *KRAS*, *NRAS*, *BRAF* and *APC*) with relative heterogeneity score.

ID	MSI status	*ATM*		*TP53*		*KRAS*		*NRAS*		*BRAF*		*APC*	
		Mutation	HS	Mutation	HS	Mutation	HS	Mutation	HS	Mutation	HS	Mutation	HS
1	MSI	K610T	60	R248Q	70	G12V	70	—	—	—	—	E1464VfsTer8	44
2	NA	E1325Stop	94	R175C	180	—	—	—	—	—	—	—	—
3	MSS	D2870H	52	—	—	G12S	114	—	—	—	—	R1450Stop	106
4	MSS	R3047Stop	88	—	—	G12D	58	—	—	—	—	R1450Stop	46
5	MSI	R337C	32	—	—	G13D	36	—	—	—	—	I1307K	156
6	MSS	P3050L	254	P278S	173	Q61H	120	—	—	—	—	—	—
7	MSI	P604S	132	R196Stop	194	G12V	140	—	—	—	—	E1286Stop	198
8	MSS	R337C	30	I254S	200	G12V	140	—	—	—	—	R1450Stop	64
9	MSS	A1309T	136	S215R	192	—	—	—	—	—	—	S1346Stop	72
10	MSS	V410A	230	—	—	—	—	—	—	—	—	—	—
11	MSS	R337H	34	—	—	—	—	—	—	—		I1311MfsTer10	200
12	NA	R2443Q	184	R273C	290	—	—	—	—	—	—	E1353FfsTer20	284
13	MSS	R337C	114	—	—	A146T	212	—	—	—	—	T1438HfsTer35	106
14	MSS	E1704D	240	—	—	—	—	—	—	—	—	E1379Stop	710
15	MSS	Q2729H	128	—	—	A146T	104	—	—	—	—	T1556NfsTer3	76
16	MSS	V410A	200	—	—	—	—	—	—	—	—	E1309DfsTer4	158
17	MSS	R337H	46	R249G	42	G12V	46	—	—	—	—	—	—
18	MSS	P604S	194	R273H	306	—	—	—	—	—	—	Q12894Stop	320
19	MSS	R2691H	52	C238Y	122	—	—	—	—	—	—	E1317Q	158
20	MSS	R337C	50	—	—	G12V	96	—	—	—	—	H1349QfsTer4	166
21	MSS	S333F	40	G266E	760	—	—	—	—	—	—	—	—
22	MSS	L1939V	44	R282W	268	—	—	—	—	—	—	—	—
23	MSS	V410A	142	I251S	98	—	—	—	—	—	—	E1547Stop	86
24	MSS	V410A	314	Y205H	207	—	—	—	—	—	—	—	—
25	MSS	R337H	108	R175H	196	G13D	190	—	—	—	—	—	—
26	MSS	S333F	290	R273C	140	—	—	—	—	—	—	—	—
27	MSS	S1691R	204	R175H	113	—	—	—	—	—	—	—	—
28	MSS	splice site 184_185 + K1992T	212 + 102	V73fs*50	90	—	—	—	—	V600E	350	—	—
29	MSI	F1928fs*9	206	R27H + R17H	340	—	—	—	—	V600E	468	—	—
30	MSS	F858L	300	V274F	66	—	—	—	—	—	—	—	—
31	MSS	R337H	20	—	—	A146T	140	—	—	—	—	—	—
32	MSS	R2912G	116	R2912G	400	—	—	G12S	120	—	—	—	—
33	MSS	G2695V	46	G245S	60	—	—	—	—	—	—	R1450Stop	50
34	MSS	V410A	132	R273C	108	G12V	—	—	—	—	—	R1450Stop	62
35	MSS	F858L	146	R282W	202	—	—	—	—	—	—	Q1291Stop	84

*Abbreviations*. HS: heterogeneity score. MSI: microsatellite instability. MSS: microsatellite stability.

### Prognostic role of ATM mutations in mCRC patients

**Figure 1:**
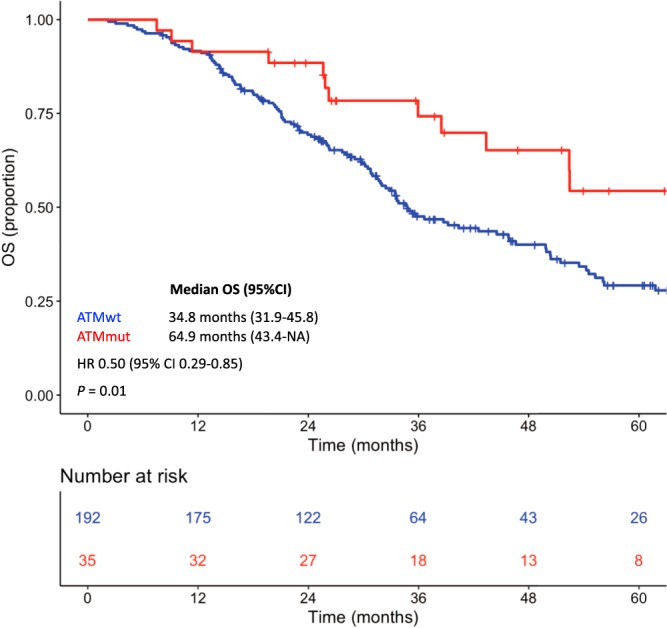
Kaplan-Meier curves for overall survival according to *ATM* mutational status. Red line indicates patients with *ATM* mutated tumors, blue line indicates patients with *ATM* wild-type tumors.

At a median follow-up of 56.6 months (95% CI, 46.3–62.1), patients with *ATM* mutated tumors showed a significantly longer median OS than patients with *ATM* wild-type tumors (64.9 versus 34.8 months; HR, 0.50; 95% CI, 0.29–0.85; *P* = 0.01) (Fig. 1). In the multivariable model (Table 3), including other covariates significantly associated with OS, the presence of *ATM* mutations confirmed its association with improved OS (HR, 0.57; 95% CI, 0.33–0.98; *P* = 0.04), along with left-sided primary tumor location (*P* = 0.005), primary tumor resection (*P* = 0.003), metachronous metastases (*P* = 0.005) and the presence of a single site of metastasis (*P* = 0.03).

**Table 3:** Univariate and multivariate analyses for overall survival.

Characteristics		Univariate analyses		Multivariable model	
		HR (95% CI)	*P*	HR (95% CI)	*P*
Age (years)	≥65 vs <65	1.60 (1.10–2.20)	**0.009**	—	0.25
Gender	Female vs Male	—	0.24	—	—
ECOG PS	1–2 vs 0	—	0.15	—	—
Primary tumor location	Right vs Left	2.00 (1.40–2.80)	**<0.001**	1.70 (1.17–2.46)	**0.005**
Primary tumor resection	No vs Yes	1.90 (1.20–2.90)	**0.005**	1.62 (1.04–2.54)	**0.03**
Synchronous mets	Yes vs No	1.80 (1.20–2.60)	**0.003**	1.76 (1.19–2.61)	**0.005**
Metastatic sites (N)	>1 vs 1	1.70 (1.20–2.40)	**0.002**	1.47 (1.03–2.08)	**0.03**
All-*RAS* status	Mut vs wt	—	0.06	—	—
*BRAF* status	Mut vs wt	2.10 (1.10–4.00)	**0.03**	—	0.09
MSI status	MSI vs MSS	—	0.16	—	—
*ATM* status	Mut vs wt	0.50 (0.29–0.85)	**0.01**	0.57 (0.33–0.98)	**0.04**

*Abbreviations*. ECOG PS: Eastern Cooperative Oncology Group Performance Status. Mets: metastases. MSI: microsatellite instability. MSS: microsatellite stable. Mut: mutated. Wt: wild-type.

**Figure 2:**
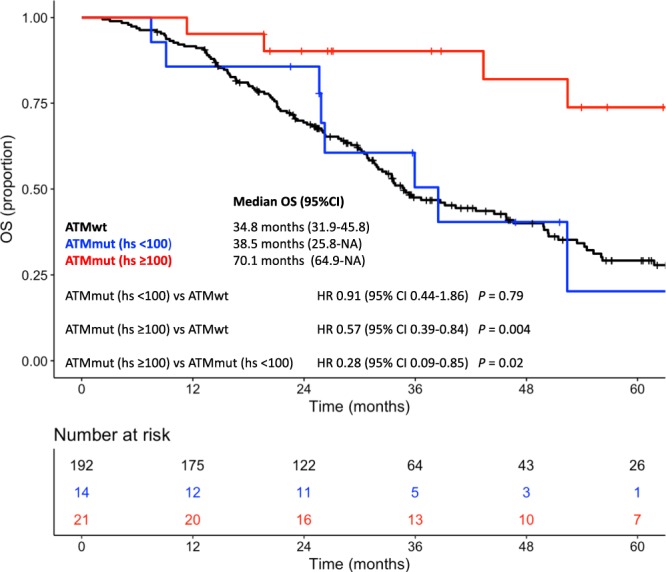
Kaplan-Meier curves for overall survival according to *ATM* mutational status and *ATM* mutational heterogeneity score. Red line indicates patients with *ATM* mutated tumors and *ATM* HS ≥ 100, blue line indicates patients with *ATM* mutated tumors and *ATM* HS < 100, black line indicated patients with *ATM* wild-type tumors. *Abbreviations*: HS: heterogeneity score.

Among patients with *ATM* mutated tumors, an HS ≥ 100 for *ATM* mutations was associated with a longer median OS compared with an HS < 100 (70.1 versus 38.5 months; HR, 0.28; 95% CI 0.09–0.85; *P* = 0.02) (Fig. 2). Therefore, when using patients with wild-type *ATM* as reference, the HR for patients with *ATM* mutated tumors and HS < 100 was 0.91 (95% CI, 0.44–1.86; *P* = 0.79), whereas it relevantly decreased for patients with *ATM* mutated tumors and HS ≥ 100 (HR, 0.57; 95% CI, 0.39–0.84; *P* = 0.004).

Of note, no prognostic significance was observed for *TP53* mutational status (*P* = 0.79), and the prognostic impact of *ATM* mutations was completely independent from the concomitant presence of *TP53* mutations (Supplementary Fig. S2 in Supplementary Information) or primary tumor sidedness (Supplementary Fig. S3 in Supplementary Information).

Finally, since we performed a massively parallel sequencing of multiple cancer-related genes, we assessed the prognostic value of the top mutated genes (i.e. those found mutated in at least 5% of samples: *ATM*, *KRAS*, *BRAF*, *NRAS*, *APC*, *PIK3CA*, *SMAD4*, *FBXW7* and *MET*) and applied the Benjamini–Hochberg procedure in order to decrease the false discovery rate, demonstrating that the *P*-value for *ATM* mutational status remained significant (*P* = 0.04) (Supplementary Table S2 in Supplementary Information).

## Discussion

Given the crucial role of ATM activity in orchestrating the DDR, relevant phenotypic spillover is awaited upon its loss. However, as a result of both biological complexity of the DDR network and heterogeneity across different studies, no conclusive clinical data are available on ATM prognostic and/or predictive impact.

In early stage CRC, low ATM expression has been previously associated with worse outcomes. In a series of 330 early CRCs, the presence of ATM expression detected by immunohistochemistry (IHC) was associated with disease-free survival and OS benefit when considering patients who underwent adjuvant treatments (N = 33)^19^. Similar results have been confirmed by a subgroup analysis of the VICTOR trial, which included stage II/III CRC patients undergoing adjuvant fluoropyrimidine-based chemotherapy^20^. Regarding the metastatic setting, a recent monocentric study showed that ATM deficiency (as primarily assessed by IHC) may be associated with improved OS following oxaliplatin-based first-line treatment, but not irinotecan-based one^21^. Discrepancy in available evidences might be related to the different prognostic role of ATM loss of function according to disease stage, similarly to what reported for MSI^22^, and the confounding effects derived from the heterogeneity of available regimens and treatment sequences used for metastatic disease.

This is the larger available study assessing the role of *ATM* mutations as prognostic biomarker in mCRC. Here, the presence of *ATM* mutations was independently associated with improved OS (adjusted HR, 0.57; 95% CI, 0.33–0.98; *P* = 0.04). These results suggest that *ATM* mutations might identify a biologically distinct disease with a survival advantage in the metastatic setting linked, at least in part, to an increased chemosensitivity. Intriguingly, patients with *ATM* mutations and an HS ≥ 100, showed the best outcomes in terms of OS. As previously described by Normanno *et al*.^17^, HS virtually corresponds to the fraction of neoplastic cells bearing a specific mutation. Specifically, an HS > 100 might reflect the loss of the wild-type allele. HS might help identifying tumors with a “functional knock-out” of ATM that lose their ability of properly coping with DNA damage. Therefore *ATM* HS should be taken into account by future studies and potentially correlated with functional data.

From a preclinical point of view, p53 is one of the most characterized ATM targets, required for G1/S cell arrest and apoptosis. Conceptually, drugs inducing high amount of DNA damage in S phase in cells with both DNA repair and G1/S–G2/M checkpoint deficiency (such as those bearing both *ATM* and *TP53* mutations) are likely to induce a mitotic catastrophe-mediated cell death^23^. However, we did not find any clinically relevant interaction between *ATM* and *TP53* mutational status in impacting on OS (Supplementary Fig. S2 in Supplementary Information). It must be pointed out that, even if ATM or CHK2 suppression preferentially sensitizes p53 deficient tumors to genotoxic drugs, a chemosensitivity status driven by ATM deficiency might occur independently from *TP53*^24^.

In addition, an enrichment of *ATM* mutations is expected in mCRC patients with right-sided tumors^18^, MSI-high^14^ or CMS1 ones^25^. In our study, the prognostic impact of *ATM* mutational status was independent from primary tumor location status (Table 3 and Supplementary Fig. S3 in Supplementary Information), even if the low number of patients with *ATM* mutations and right-sided mCRC highlights the need of larger datasets to specifically assess the impact of DDR alterations according to primary tumor location or disease subtypes.

Our study has some limitations. For instance, despite the strong rationale making *ATM* mutational status a candidate biomarker of response to oxaliplatin and/or irinotecan^26–29^, we have not considered response rate or progression-free survival because of the heterogeneity of treatment regimens as per standard practice (fluoropyrimidine monotherapy, doublet or triplet chemotherapy regimens associated or not with anti-VEGF or anti-EGFR). Of course, an integrated assessment of both protein expression and mutational status would be necessary for identifying all tumors with clinically relevant *ATM* loss of function. In fact, other mechanisms might account for ATM reduced activity, such as low expression due to promoter methylation^30^. Indeed, a comprehensive assessment of the DDR network on a proteomic scale is expected to reach the best accuracy for predicting chemosensitivity.

Beyond being a sole biomarker of chemosensitivity, *ATM* mutations might predict response to DDR-targeting agents paralleling recent achievements in other clinical settings, such as castration-resistant prostate cancer (CRPC). Indeed, in the TOPARP-A phase II trial CRPC patients bearing alterations in homologous recombination repair genes displayed a high response rate to the PARP inhibitor olaparib (including 4 out of 5 patients with tumors bearing *ATM* mutations)^31^. Similar therapeutic approach would be backed by a strong preclinical rationale also in CRC^32^. The reader is referred to Choi *et al*.^33^ for reviewing potential synthetic lethality strategies (*e*.*g* PARP1 or ATR inhibitors) in ATM deficient tumors.

In conclusion, our study suggests that *ATM* mutations with high HS might characterize a subset of mCRC at better prognosis. From this background, further investigations are needed to cover crucial unresolved issues such as the assessment of functional relevance of specific *ATM* mutations and their predictive role upon specific DNA damaging and/or DDR-targeting agents. Indeed, synthetic lethality strategies might be preferentially used in ATM deficient tumors, while ATM proficient tumors might be sensitized to conventional therapies by ATM inhibitors^34^. Thus, *ATM* mutational status could enter the clinical decision-taking process in parallel with the development of specific targeted strategies.

## Supplementary information

Supplementary Information

## Data Availability

The datasets generated during and/or analyzed during the current study are available from the corresponding author on reasonable request.
