# Supplementary material for: Prognostic impact of ATM mutations in patients with metastatic colorectal cancer
Source: Sci Rep. 2019 Feb 27;9:2858. doi: 10.1038/s41598-019-39525-3 (PMC6393680; doi:10.1038/s41598-019-39525-3)

SUPPLEMENTARY INFORMATION

Prognostic impact of *ATM* mutations in patients with metastatic colorectal cancer

Giovanni Randon, Giovanni Fucà, Daniele Rossini, Alessandra Raimondi, Filippo Pagani, Federica Perrone, Elena Tamborini, Adele Busico, Giorgia Peverelli, Federica Morano, Monica Niger, Maria Antista, Salvatore Corallo, Serena Saggio, Beatrice Borelli, Gemma Zucchelli, Massimo Milione, Giancarlo Pruneri, Maria Di Bartolomeo, Alfredo Falcone, Filippo de Braud, Chiara Cremolini and Filippo Pietrantonio

Supplementary Methods

Supplementary Table S1

Supplementary Table S2

Supplementary Figure S1

Supplementary Figure S2

Supplementary Figure S3

Supplementary Methods

Formalin-fixed, paraffin-embedded colorectal cancer specimens were sliced in 5um sections and manually microdissected to isolate the tumor area carrying the highest percentage of neoplastic cells identified by a pathologist on hematoxylin-eosin. A minimal tumour percentage of 10% was required; average tumor percentage was 70% (range 10%-90%) and no difference of percentages was observed between *ATM* mutated and wild-type samples. Sample were treated with xylene and 100% ethanol to remove paraffin and then DNA was isolated using the GeneRead DNA FFPE kit (Qiagen, Hilden, Germany, http://www.qiagen.com Cat. n. 180134). DNA amount and quality were identified using Nano Drop platform and Qbit dsDNA BR kit (Thermofisher) following the manufacturer's instructions.

Molecular characterization of samples was planned through next generation sequencing (NGS). Genomic DNA (10 ng) was profiled using the Ion AmpliSeq™ Cancer Hotspot Panel (ThermoFisher) that is designed to amplify 50 gene hotspot regions (207 amplicons) covering about 2800 COSMIC mutations in oncogenes and tumor suppressor genes commonly mutated in human cancers (ABL1, AKT1, ALK, APC, ATM, BRAF, CDH1, CDKN2A, CSF1R, CTNNB1, EGFR, ERBB2, ERBB4, EZH2, FBXW7, FGFR1, FGFR2, FGFR3, FLT3, GNA11, GNAS, GNAQ, HNF1A, HRAS, IDH1, IDH2, JAK2, JAK3, KDR/ VEGFR2, KIT, KRAS, MET, MLH1, MPL, NOTCH1, NPM1, NRAS, PDGFRA, PIK3CA, PTEN, PTPN11, RB1, RET, SMAD4, SMARCB1, SMO, SRC, STK11, TP53, VHL). The Ion AmpliSeq Library Kit 2.0 (Thermo Fisher) was used to amplify DNA according to the manufacturer's instructions (MAN0006735). The amplified libraries were evaluated by size and quality assessed using the Agilent Bio Analyzer DNA High Sensitivity Kit (Agilent Technologies) and quantified using the Qbit dsDNA HS kit (ThermoFisher). Template preparation and chip loading was performed using the Ion Chef System and sequencing by the Ion Torrent Personal Genome Machine™ system (ThermoFisher) using Ion 318 chips and the Ion PGM™ Hi-Q™ View Chef Kit, according to the manufacturer's instructions.

Data were processed using the Torrent Suite™ to generate sequence reads, alignment of the reads on the reference genome Hg19, trim adapter sequences, filter and remove poor signal-profile reads. The variant calling from sequencing data was generated by using the Variant Caller plugin: bases with low phred scores (e.g. Q < 20) were filtered out as part of quality control (QC) procedures and the variants with VAF (variant allele frequency) <5% were not reported. For all sample, the average base coverage depth was at least 1500x and uniformity at least 97%.

The resulting variants were annotated using the Ensemble Variant Effect Predictor pipeline, Ion Reporter™ analysis software, COSMIC database, dbSNP database, ClinVar db of the National Center for Biotechnology Information (http://www.ncbi.nlm.nih.gov/clinvar/) and, then, examined by Broad Institute's Integrative Genomics Viewer (IGV) tool to test their level of quality and to confirm the variant presence on both "+" and "-" strand. We reported all identified variants and we excluded synonymous mutations and the variants with MAF greater than 0.01 in 1000 genomes combined population (polymorphisms).

Supplementary Table S1. Exposure to specific drugs approved for mCRC, and the number of treatment lines received, overall and according to *ATM* mutational status.

**Characteristic**	**Overall population (N = 227) N (%)**	***ATM* mutated (N = 35) N (%)**	***ATM* wild-type (N= 192) N (%)**
**Specific drug exposure**			
**Fluoropyrimidines**	224 (99)	34 (97)	190 (99)
**Oxaliplatin**	208 (92)	33 (94)	175 (91)
**Irinotecan**	190 (84)	31 (88)	159 (83)
**Bevacizumab/aflibercept**	170 (75)	28 (80)	142 (74)
**Cetuximab/panitumumab***	121 (53)	18 (51)	103 (54)
**Regorafenib**	53 (23)	5 (14)	48 (25)
**Trifluridine/tipiracil**	21 (9)	4 (11)	17 (9)
**Number of treatment lines**			
**1**	41 (18)	7 (20)	34 (18)
**2**	56 (25)	8 (23)	48 (25)
**3**	44 (19)	6 (17)	38 (20)
**>3**	86 (38)	14 (40)	72 (37)

*If *RAS* wild-type as per local assessment.

Supplementary Table S2. Prognostic value in terms of overall survival of top mutated genes (i.e. those mutated in at least 5% of samples) adjusted for the false discovery rate due to multiple testing.

**Gene**	**N (%)**	**HR (95% CI) for OS**	***P***	**Adjusted *P* (Benjamini and Hochberg method)**
All-RAS	100 (44)	1.37 (0.98-1.92)	0.06	0.12
BRAF	13 (6)	2.10 (1.10-4.00)	0.03	0.08
ATM	35 (15)	0.50 (0.29-0.85)	**0.01**	**0.04**
APC	105 (46)	0.99 (0.71-1.39)	0.96	0.96
PIK3CA	32 (14)	1.78 (1.14-2.79)	**0.01**	**0.04**
SMAD4	29 (12)	0.90 (0.54-1.51)	0.70	0.93
FBXW7	18 (8)	1.17 (0.65-2.12)	0.61	0.93
MET	15 (7)	0.95 (0.51-1.77)	0.87	0.96

Abbreviations. HR: hazard ratio. OS: overall survival.

Supplementary Figure S1. Study's consort flow diagram.

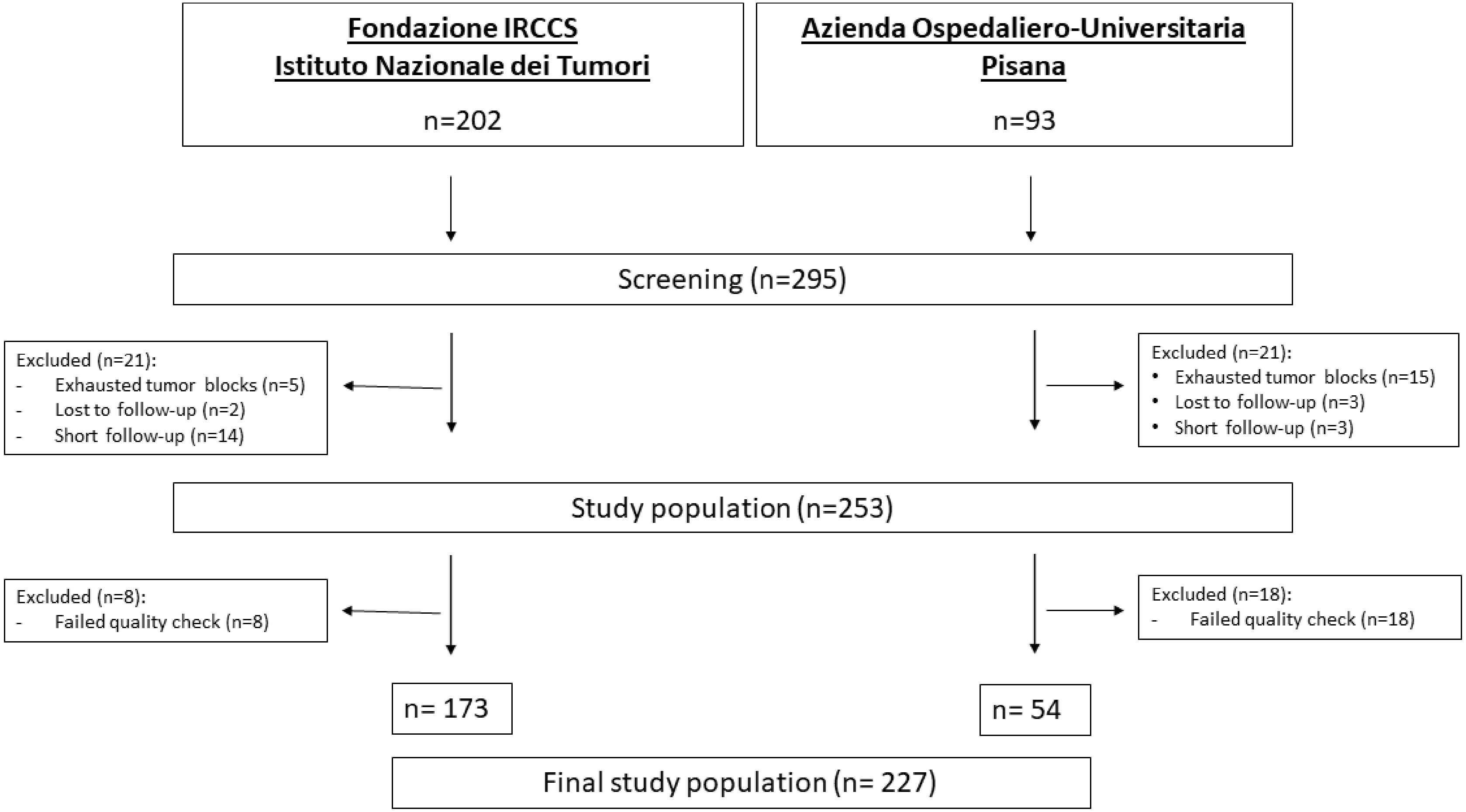

Supplementary Figure S2. Kaplan-Meier curves for overall survival according to *ATM* and *TP53* mutational status.

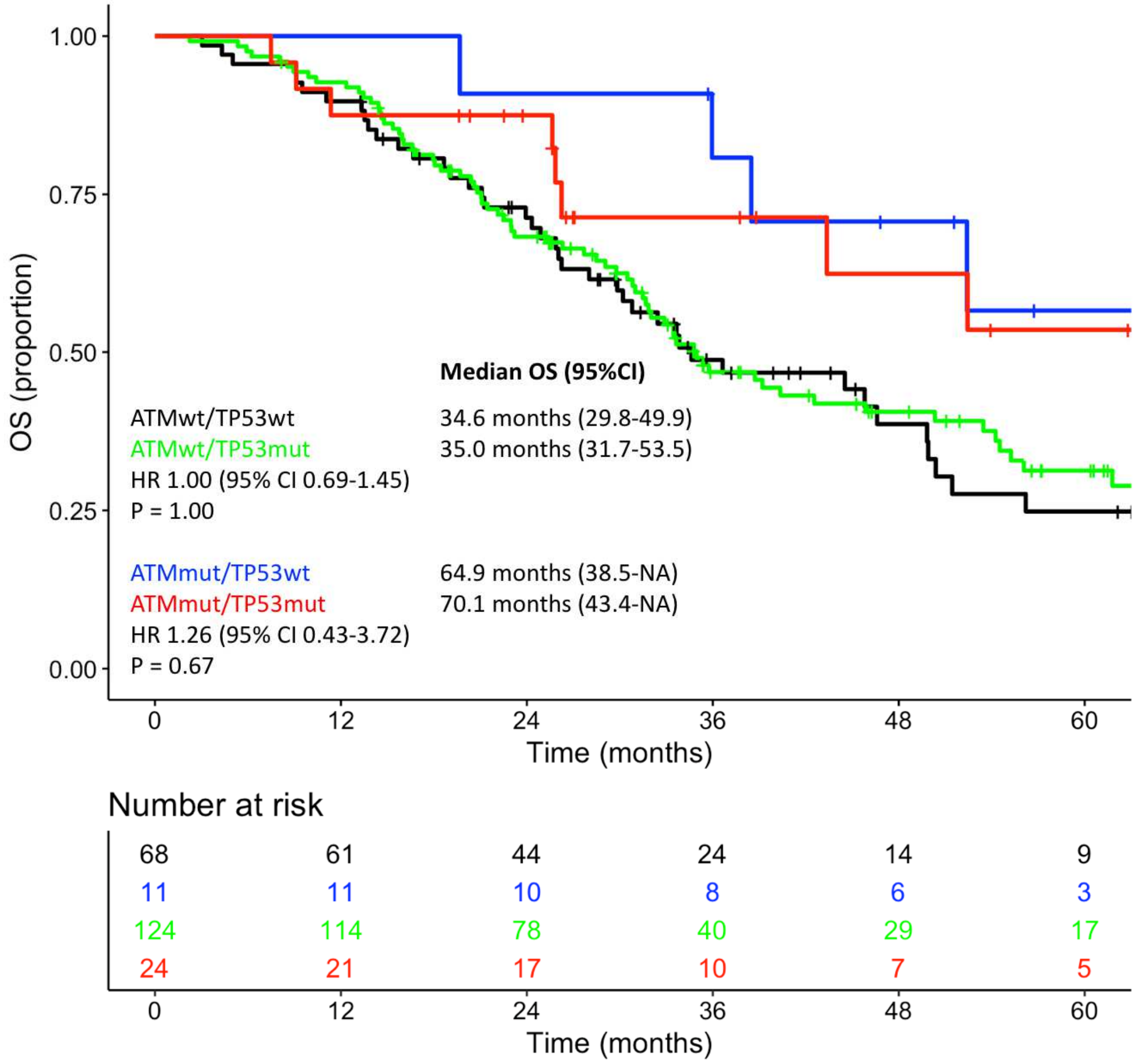

Supplementary Figure S3. Kaplan-Meier curves for overall survival according to *ATM* mutational status and primary tumor sidedness.

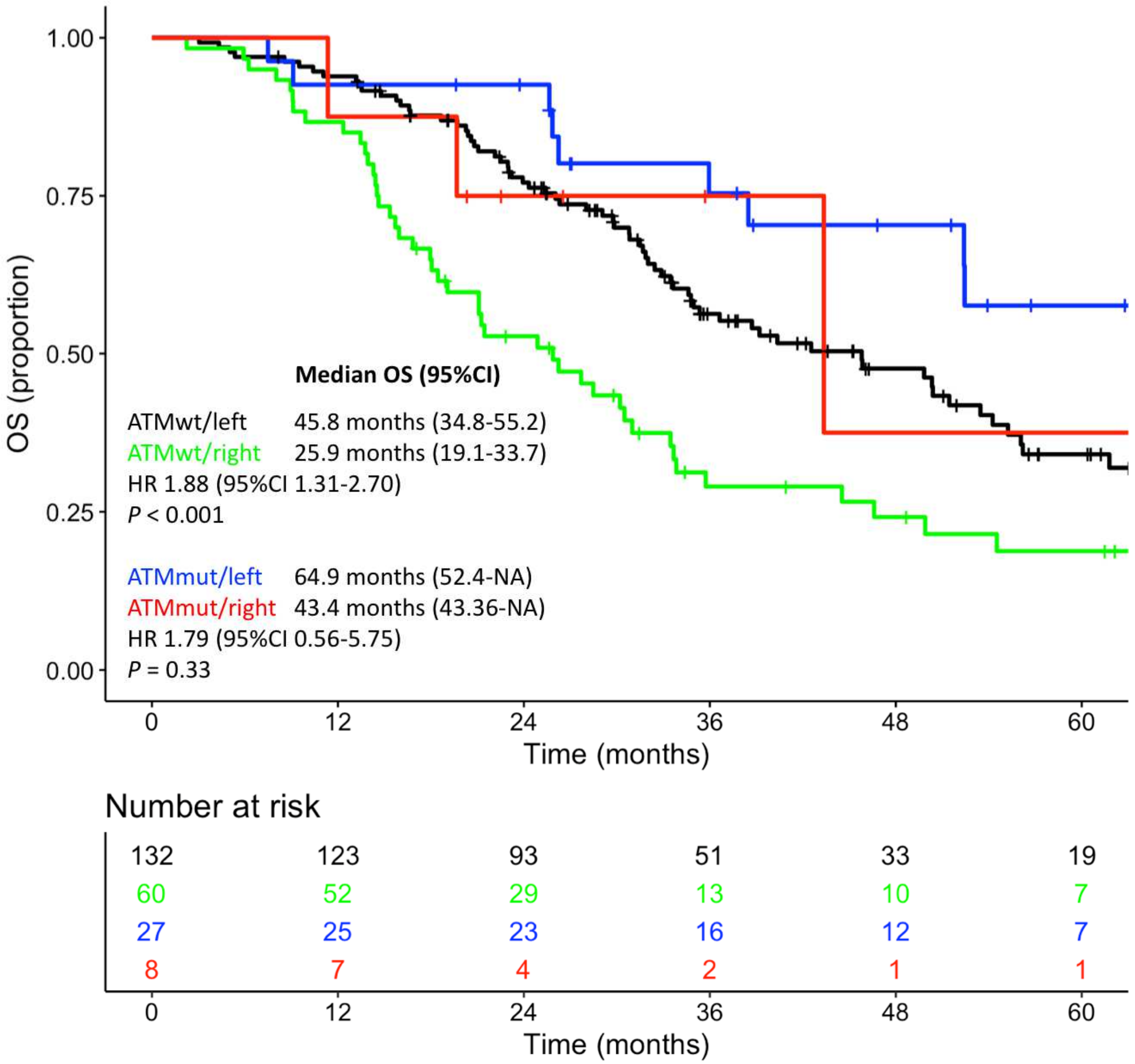

Supplement: Supplementary file 1 — Supplementary Information [file 41598_2019_39525_MOESM1_ESM.pdf]
